# Seroprevalence of *Toxoplasma gondii* infection in women with a gynecological tumor living in eastern China

**DOI:** 10.7717/peerj.14569

**Published:** 2022-12-15

**Authors:** Zhongjun Wang, Tingting Qu, Huiyang Qi, Shuchao Zhao, Hailei Shi, Wenye Bai, Yang Yu, Xiao Wu, Peng Zhao

**Affiliations:** 1 Department of Clinical Laboratory, The Affiliated Hospital of Qingdao University, Qingdao, Shandong, China; 2 Department of Pathology, The Affiliated Hospital of Qingdao University, Qingdao, Shandong, China; 3 Department of Hepatobilary and Surgery Pancreatic Surgery, The Affiliated Hospital of Qingdao University, Qingdao, Shandong, China; 4 Department of Clinical Laboratory, Qingdao Women and Children’s Hospital, Qingdao, Shandong, China

**Keywords:** *Toxoplasma gondii*, Gynecological tumor, Seroprevalence

## Abstract

The association between *Toxoplasma gondii (T. gondii*) infection and malignancy has attracted increased attention in recent years, but little is known of *T. gondii* infection among women diagnosed with a gynecological tumor (GT) in China. We conducted a case-control study involving 460 women diagnosed with a GT and 460 age-matched healthy controls (HCs) to estimate the infection process of *T. gondii* and understand the risk factors of *T. gondii* infection in patients with a GT. Levels of anti-*T. gondii* IgG and IgM were measured by enzyme-linked immunoassays every 12 months. After a median follow-up time of 4.3 years (range 4 to 5 years), 55/460 (11.96%) patients with a GT and 15/460 (3.26%) HCs were seroprevalence for *T. gondii* antibodies, respectively (*P* = 0.001). IgG antibodies against *T. gondii* were found in 54 GT patients (11.74%) and 15 HCs (3.26%), respectively (*P* = 0.001). The seroprevalence of *T. gondii* IgM antibodies was similar in patients with a GT and with HCs (2.83% *vs* 1.3%, *P* = 0.105). Multivariate stepwise logistic regression analysis revealed contact with cats (OR, 6.67; 95% CI [2.89–10.75]; *P* = 0.001), exposure to soil (OR, 2.16; 95% CI [1.14–4.10]; *P* = 0.019), being a farm-worker (OR, 4.17; 95% CI [1.20–11.49]; *P* = 0.006) and history of chemotherapy (OR, 3.16; 95% CI [1.56–6.45]; *P* = 0.001) to be independent risk factors for *T. gondii* infection. Women with an ovarian cancer or endometrial cancer had higher *T. gondii* seroprevalence than that of HCs. Moreover, *T. gondii* infection in patients with a GT mostly acquired within two years of diagnosis, but the infection in healthy controls had no obvious time characteristics. Here, we demonstrated that *T. gondii* infection is significantly higher in patients with a GT (especially in women with an ovarian tumor) compared to HCs. Thus, infection with this parasite should be avoided in patients with a GT, and the causal relationship between *T. gondii* and GTs should be studied in detail.

## Introduction

*Toxoplasma gondii (T. gondii)* is an intracellular parasitic protozoan that causes toxoplasmosis and chronically infects nearly one-third of humans worldwide (Elmore et al., 2010). *T. gondii* has three infective forms: tachyzoite, bradyzoites within tissue cysts, and sporozoites in sporulated oocysts (Mévélec, Lakhrif & Dimier-Poisson, 2020). Efficient immunity (*via* cluster of differentiation CD4^+^ cells, CD8^+^ T cells, and macrophages) reduces cyst formation (Hamie et al., 2021). Hence, an acute infection of *T. gondii* is usually self-limiting and asymptomatic in immunocompetent individuals. However, in immunosuppressed patients, cysts can localize, proliferate readily, and lead to *T. gondii* infection (Dard et al., 2018; Tian et al., 2017). Thus, in immunosuppressed patients (*e.g*., recipients of organ transplants and cancer patients), *T. gondii* can cause fatal encephalitis, myocarditis, pneumonitis, chorioretinitis, or generalized lymphadenopathy (Alvarado-Esquivel et al., 2010; Dard et al., 2018).

There is increasing interest in exploring the causation between *T. gondii* infection and cancer (Caner, 2021; Liu et al., 2019). Thomas et al. (2012) demonstrated that toxoplasmosis might cause brain cancer. Another study reported a latent relationship between *T. gondii* and oral cancer (Zhou et al., 2018). Moreover, Kalantari et al. (2017) detected *T. gondii* DNA from formalin-fixed, paraffin-embedded breast cancer tissues, which provided direct evidence for *T. gondii* infection and breast cancer. These data suggest that *T. gondii* infection is a significant phenomenon in cancer patients. Gynecological tumors (GT) can grow in any part of the pelvic cavity in women. The classification of gynecological tumors comprises benign tumors (uterine leiomyoma and mature ovarian teratomas), malignant tumors (cervical cancer, endometrial cancer, and ovarian cancer), and borderline tumors. Cong et al. (2015b) showed that women with ovarian cancer in China harbored high seroprevalence of *T. gondii*.

Cats are the only definitive host for *T. gondii* (Harker, Ueno & Lodoen, 2015). Humans can be infected with *T. gondii* by having contact with cats, eating undercooked meat, or having regular contact with soil harboring *T. gondii* (Elmore et al., 2010; Tian et al., 2017; Zhou et al., 2019). Studies conducted by Yu and Zhou showed *T. gondii* to be common in cancer patients in eastern China, and that blood transfusion and chemotherapy could promote the spread of this parasite (Zhou et al., 2018; Yu et al., 2020). Epidemiological investigation of *T. gondii* in cancer patients has been undertaken in recent years (Cong et al., 2015b; Yu et al., 2020). However, the seroprevalence of *T. gondii* in patients with a benign GT in eastern China is not known, nor indeed the potential risk factors of *T. gondii* infection in such patients. The current study was conducted to fill these knowledge gaps.

## Methods

### Ethical approval of the study protocol

The study protocol was approved (QYFY WZLL 26823) by the Ethics Committee of the Affiliated Hospital of Qingdao University (Qingdao, China). All patients/guardians provided written informed consent.

### Study cohort

From April 2016 to June 2018, 460 women diagnosed with a primary GT were recruited from the Affiliated Hospital of Qingdao University. An identical number of healthy controls (HCs), with similar age and residence, were recruited. No patients were seropositive for *T. gondii* before being recruited. Study participants were followed up until June 2022, and the data concerning *T. gondii* infection and patient survival were collected.

### Sample collection

Approximately 5 mL of venous blood was collected from participants once a year. Blood samples were left for 2 h at room temperature to allow clotting, and then centrifuged at 3,000 rpm for 10 min at room temperature. Serum was collected in 2-mL Eppendorf tubes and stored at −80 °C until analyses.

### Sociodemographic and clinical data

Sociodemographic data (including age, residence area, and occupation) were collected from participants. Lifestyle variables, including contact with animals (cats, pigs, and/or dogs), consumption of undercooked meat, of raw vegetables or fruits, contact with soil, and source of drinking water, were obtained *via* a structured questionnaire (Yu et al., 2020). Clinical data were collected from the participant’s medical records.

### Serological assay

Serum samples were tested for IgG and IgM antibodies against *T. gondii* using enzyme-linked immunosorbent assay (ELISA) kits (Kanghua Bio, Beijing, China), as previously described (Yu et al., 2020). Briefly, serum (10 μL) and sample diluent (100 μL) were mixed and dropped into a well on the plate coated with *T. gondii* antigen. The plate was then incubated in the dark and allowed for 20 min at 37 °C. Fifty microliters of horseradish peroxidase-conjugated enzyme was added to each well, after washing twice with distilled water. Next, solution A (50 µL) and solution B (50 µL) were added to the microtiter plate and incubation allowed for 10 min at 37 °C, followed by washing twice with distilled water. The reaction was stopped by addition of termination solution (50 μL), and the optical density (OD) was measured using an automated microplate reader (Infinite f200; Tecan, Melbourne, Australia) at 450 nm. The cutoff value was calculated to be 2.1-times the mean OD for the negative control, and results equal to or greater than the cutoff value were considered positive. Positive and negative control sera were included in each plate. Samples from patients and HCs were mixed randomly.

### Statistical analyses

The PASS 11.0 statistical package was used to calculate the sample size and the power of the study was 95%. Results were analyzed using SPSS 19.0 (IBM, Armonk, NY, USA). The categorical variables associated with the seroprevalence of *T. gondii* antibodies were explored with the chi-square test or Fisher’s exact test. Variables were included in a multivariate stepwise logistic regression analysis if *P* < 0.05 in the univariate analysis. The adjusted odds ratio (OR) and 95% confidence interval (CI) were calculated to identify the independent risk factors for *T. gondii* infection. *P* < 0.05 was considered to be statistically significant.

## Results

### Epidemiology and risk factors for patients with a GT and *T. gondii* infection

A total of 920 participants (460 patients with a GT and 460 HCs) were evaluated. After a median follow-up time of 4.3 years (range 4 to 5 years), the overall seroprevalence of *T. gondii* antibodies in patients with a GT and HCs was 55/460 (11.96%) and 15/460 (3.26%), respectively (*P* = 0.001). Moreover, IgG antibodies against *T. gondii* were detected in 54 patients with a GT (11.74%) compared to 15 HCs (3.26%) (*P* = 0.001). Thirteen patients with a GT (2.83%) and six (1.30%) HCs were positive for IgM antibodies (*P* = 0.105). In addition, one patient had a single positive IgM for *T. gondii* (Table 1). The sociodemographic and clinical data of patients with a GT and HCs (including age, residence area, and occupation), and data for lifestyle and primary disease, are shown in Table 2. Univariate analysis showed some sociodemographic and clinical variables with a *P* < 0.05, including: contact with cats, exposure to soil, being a farm-worker and history of chemotherapy (Table 2). Multivariate stepwise logistic regression analysis revealed contact with cats (OR, 6.67; 95% CI [2.89–10.75]; *P* = 0.001), exposure to soil (OR, 2.16; 95% CI [1.14–4.10]; *P* = 0.019), being a farm-worker (OR, 4.17; 95% CI [1.20–11.49]; *P* = 0.006) and history of chemotherapy (OR, 3.16; 95% CI [1.56–6.45]; *P* = 0.001) to be independent risk factors for *T. gondii* infection (Table 3).

**Table 1:** Combined IgG and IgM anti- *T. gondii* antibody seroprevalence in patients with gynecological tumor and healthy controls.

Sero-reaction	Patients with a gynecological tumor (*n* = 460)		Healthy controls (*n* = 460)		Patients with a gynecological tumor *vs* Healthy controls
	No. positive	%	No. positive	%	*P*
IgG	54	11.74	15	3.26	0.001
IgM	13	2.83	6	1.3	0.105
IgG^+^/IgM^+^	12	2.61	6	1.3	0.153
IgG^+^/IgM^−^	42	9.13	9	1.96	0.001
IgG^−^/IgM^+^	1	0.22	0	0	1
Total	55	11.96	15	3.26	0.001

**Table 2:** Seroprevalence of *T. gondii* infection in patients with gynecological tumor and control subjects in eastern China.

Characteristic	Patients with a gynecological tumor (*n* = 460)				Healthy controls (*n* = 460)			
	Prevalence of *T. gondii* infection				Prevalence of *T. gondii* infection			
	No. tested	No. positive	%	*P*	No. tested	No. positive	%	*P*
Age (years)								
≤30	95	3	3.16%	0.101	65	5	7.69%	0.216*
31–50	112	11	9.82%		123	5	4.07%	
50–70	192	33	17.19%		237	5	2.11%	
>71	61	8	13.11%		35	0	0.00%	
Residence area								
Urban	257	32	12.45%	0.71	245	5	2.04%	0.31
Rural	203	23	11.33%		215	10	4.65%	
Contact with cats								
Yes	142	38	26.76%	0.001	119	11	9.24%	0.001*
No	318	17	5.35%		341	4	1.17%	
Contact with dogs								
Yes	209	26	12.44%	0.77	108	2	1.85%	0.54*
No	251	29	11.55%		352	13	3.69%	
Contact with pigs								
Yes	74	7	9.46%	0.47	91	3	3.30%	1*
No	386	48	12.44%		369	12	3.25%	
Consumption of raw/undercooked meat								
Yes	109	10	9.17%	0.31	76	8	10.53%	0.001*
No	351	45	12.82%		384	7	1.82%	
Consumption of raw vegetables								
Yes	79	7	8.86%	0.35	209	10	4.78%	0.09
No	381	48	12.60%		251	5	1.99%	
Exposure to soil								
Yes	188	30	15.96%	0.03	144	7	4.86%	0.26*
No	272	25	9.19%		316	8	2.53%	
Source of drinking water								
Tap	352	43	17.00%	0.76	271	12	4.43%	0.11
River	108	12	11.11%		189	3	1.59%	
Occupation								
Farmer	280	50	17.86%	0.001	304	15	4.93%	0.005
Worker	180	5	2.78%		156	0	0.00%	
History of abortion								
Yes	76	10	13.16%	0.73	117	8	6.84%	0.03*
No	384	45	11.72%		343	7	2.04%	
History of chemotherapy								
Yes	186	51	27.40%	0.001				
No	274	4	1.46%					
History of blood transfusion								
Yes	122	10	8.20%	0.14				
No	338	45	13.31%					

**Note:**

* Fisher’s exact test.

**Table 3:** Multivariable analysis of patients with gynecological tumor and healthy controls and the association of characteristics with *T. gondii* infection.

Characteristic		Adjusted odds ratio^a^	95% CI^b^	*P*
Contact with cats	Yes *vs* No	6.67	[2.89–10.75]	0.001
Exposure to soil	Yes *vs* No	2.16	[1.14–4.10]	0.019
Occupation	Farmer *vs* worker	4.17	[1.20–11.49]	0.006
History of chemotherapy	Yes *vs* No	3.16	[1.56–6.45]	0.001

**Notes:**

a Adjusted by age.

b Confidence interval.

### Seroprevalence of *T. gondii* antibodies among patients with a GT

**Figure 1:**
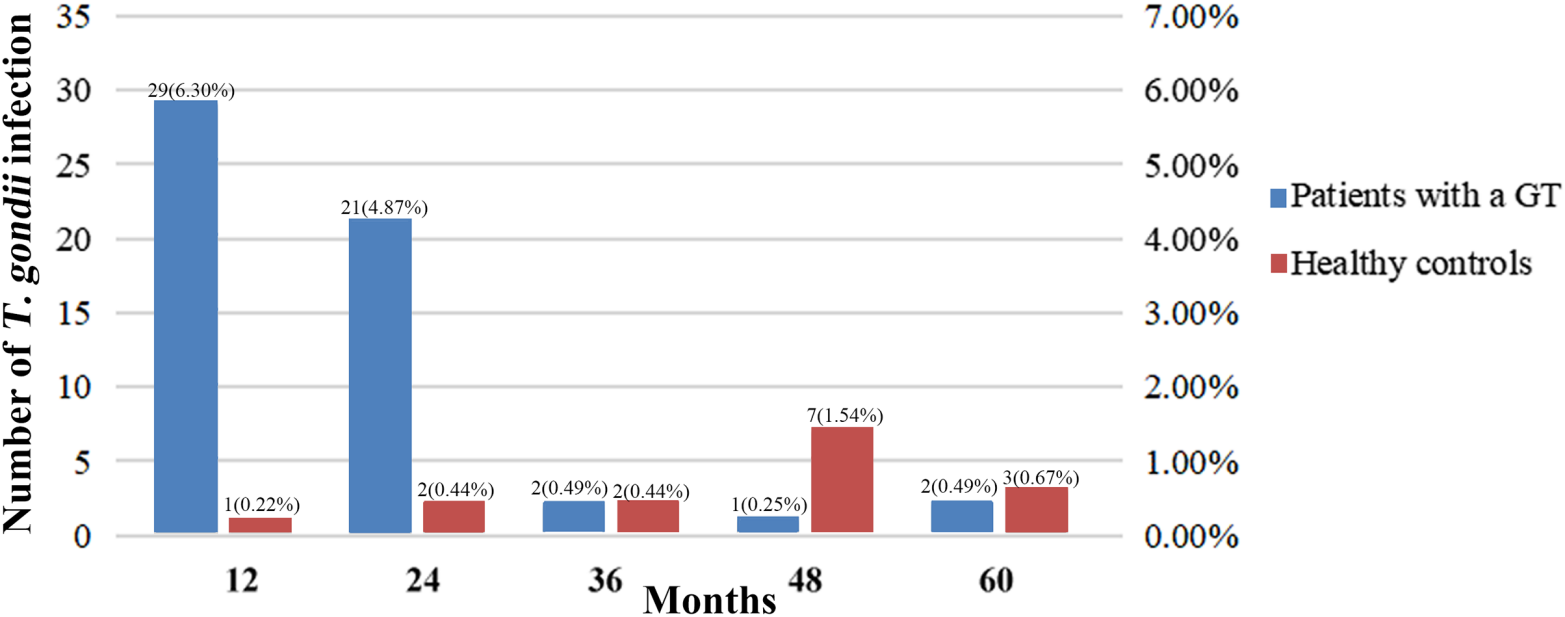
Comparison between studies on *T. gondii* serostatus and follow-up time.

The distribution of the seroprevalence of *T. gondii* antibodies according to the histology types of GTs is presented in Table 4. Women with ovarian cancer had the highest prevalence of *T. gondii* antibodies (26.79%), followed by women with endometrial cancer (24.24%) and ovarian mucinous cystadenoma (14.81%) (*P* < 0.05). In addition, 21 patients with malignant GTs died, but no patients died from toxoplasmosis. The acquired infection of *T. gondii* in patients with GTs mostly occurred within 2 years of diagnosis, while the infection in healthy controls had no obvious time characteristics (Fig. 1).

**Table 4:** Clinical diagnosis and seroprevalence of *T. gondii* in patients with gynecological tumor in eastern China.

Clinical diagnosis	No. tested	No. positive	%	*P* ^a^
Gynecological tumor	460	55	11.96%	0.001
Ovarian cancer	112	30	26.79%	0.001
Endometrial cancer	66	16	24.24%	0.001
Ovarian mucinous cystadenoma	27	4	14.81%	0.016*
Cervical squamous cell carcinoma	86	5	5.81%	0.22*
Ovarian cystic mature teratoma	109	0	0.00%	0.09*
Uterine leiomyoma	43	0	0.00%	0.63*
Ovarian borderline tumor	17	0	0.00%	0.56*

**Notes:**

a As compared with 3.26% seroprevalence of anti-*T. gondii* antibodies in controls (15/460).

* Fisher exact test were used.

## Discussion

*T. gondii* is a protozoan parasite that infects warm-blooded animals, including cats and humans (EI-Kady et al., 2022). In immunocompetent individuals, *T. gondii* infection is often asymptomatic or an influenza-like illness (Zhou et al., 2011), but it can be life-threatening in immunocompromised patients (*e.g*., patients with cancer or those who have undergone solid-organ transplantation) (Dard et al., 2018; Pinto et al., 2020; Thirugnanam, Rout & Gnanasekar, 2013). *T. gondii* bradyzoites tend to accumulate in the myocardium rather than in other tissues, so the seroprevalence of *T. gondii* antibodies tends to be significantly higher in people who have undergone heart transplants or heart-lung transplants than in people who have undergone transplantation of other solid organs (Fernandez-Sabe et al., 2011; Pinto et al., 2020). The potential relationship between *T. gondii* infection and ovarian cancer has been reported (Cong et al., 2015b; Qian, Shen & Wang, 2019). However, data on *T. gondii* infection is limited in patients with a benign GT living in eastern China. Assessment of serological status and the risk factors for *T. gondii* transmission could help to prevent death due to disseminated toxoplasmosis in a GT.

Herein, we demonstrate that the seroprevalence of *T. gondii* antibodies was significantly higher in patients with a GT (11.96%, 55/460) compared to HCs (3.26%, 15/460) at the end of the follow-up period. Malignant tumors might enhance cytolytic activity and lead to diminished secretion of anti-inflammatory cytokines (Scerra et al., 2013). The immune system could be weakened further by treatment with immunosuppressive agents after anti-cancer treatment, so patients with cancer have a weakened defense against *T. gondii* infection (Ali et al., 2019). In additional, patients with a GT harbored a higher seroprevalence of *T. gondii* IgM antibodies compared to HCs. Serological methods, such as ELISAs and immunofluorescence assays, are the most effective ways to screen for *T. gondii* infection (Dhakal et al., 2015). IgM antibodies against *T. gondii* are markers for acute *T. gondii* infection, but a single positive IgM result for *T. gondii* usually denotes a chronic infection or a false-positive result (Dhakal et al., 2015). In the present study, one sample had a single positive IgM result for *T. gondii*, and a definitive diagnosis of toxoplasmosis was made for this sample. The reasons why patients with a GT are inclined to develop a single positive IgM result for *T. gondii* have not been elucidated. Sulfamethoxazole is a common anti-parasitic drug that helps to prevent *T. gondii* infection and to reverse antibody positivity (Dard et al., 2018; Zhou et al., 2019). Moreover, patients with a GT are immunodeficient and may be unable to produce sufficient IgG to prevent parasite transmission. Therefore, a single positive IgM result for *T. gondii* could be used as an index of *T. gondii* infection for patients with a GT (Zhou et al., 2019). As stated above, a single positive IgM result for *T. gondii* is often regarded as a false-positive for *T. gondii*, and clinicians might not take measures for such patients. Hence, clinicians should pay attention to the hidden role of a single positive IgM result for *T. gondii* in patients with a GT.

Carrying out farm-work was a risk factor for *T. gondii* infection in patients with a GT. However, for HCs, contact with a cat and consumption of raw meat are also risk factors. In addition, infection with *T. gondii* can be a cause of miscarriage in women. This result is consistent with observations reported by Cong et al. (2015a) showing that cats are the definitive hosts of *T. gondii*. Oocysts shed by cats infected with *T. gondii* can be washed into rivers *via* rainfall (Deng et al., 2021; López Ureña et al., 2022; Zhou et al., 2018), and stored in silt. Development in rural areas is relatively poor, and sanitary conditions are underdeveloped. People from countryside areas are usually farmers in contact with stray cats and exposed to contaminated soil. Therefore, farmers are at a high risk of exposure to *T. gondii* (Yu et al., 2020). *T. gondii* infection is less frequent in immunocompetent individuals than in cancer patients. Therefore, the risk factors for *T. gondii* might differ between cancer patients and healthy individuals. Hence, farmers suffering from cancer must be made aware of the risk of *T. gondii* infection. Moreover, in women with a GT living in countryside areas, measures are needed to reduce the risk of *T. gondii* infection.

Interestingly, patients with an ovarian mucinous cystadenoma (14.81%), rather than an ovarian cystic mature teratoma (0%), were more susceptible to *T. gondii*. Moreover, patients with ovarian cancer had the highest seroprevalence of *T. gondii* antibodies (26.79%), which is similar to the figure reported among 112 patients with ovarian cancer in Jiangsu, China (27.68%) (Qian, Shen & Wang, 2019). These results clearly showed that toxoplasmosis is associated with ovarian cancer. However, whether cancer increases the risk of infection by *T. gondii* or whether toxoplasmosis aids treatment of cancer is not known. Some studies have demonstrated that *T.gondii* might be a promising therapeutic treatment for cancer (Wang et al., 2022; Zhu et al., 2021). Transcriptomic analyses of *T. gondii*-infected mice and *in vitro* cell cultures have suggested that *T. gondii* may suppress carcinogenesis in host cells (Wang et al., 2022). A study reported by Zhu et al. (2021) showed that combined *T. gondii* and anti-PD-L1 therapy significantly arrested melanoma and lung adenocarcinoma tumor growth in mice models. Mechanically, intratumoral inoculation of the ΔGRA17 mutant *T. gondii* strain can activate CD4^+^ T cells, CD8^+^ T cells and NK cells and decrease the expression of PD-1 in CD8^+^ T cells of hosts to improve immunological response to anti-cancer therapy. These findings suggest that intratumoral injection of *T. gondii* may activate host immune cells to kill tumor cells. Here, we conducted a prospective study in that no volunteers were infected with *T. gondii* when they were recruited. Our results showed that cancer increases the risk of *T. gondii* infection, and patients with ovarian cancer were more commonly infected with *T. gondii*. Benign tumors have received less attention compared with malignant tumors. In addition, concerns regarding toxoplasmosis are limited for patients with a benign GT, so the causation of *T. gondii* infection and ovarian tumor malignant progression should be studied further.

The seroprevalence of *T. gondii* increases with age in healthy individuals due to a greater exposure to *T. gondii* with time (Imam et al., 2017; Nowakowska et al., 2014). However, in patients with a malignant tumor, *T. gondii* infection is more prevalent in younger groups (Yu et al., 2020; Zhou et al., 2019). This phenomenon may occur because patients with a malignant tumor are immunosuppressed, and young patients may have weak immunity against *T. gondii* infection (Cong et al., 2015b). But most of these studies were retrospective or cross-sectional. Here, we conducted a prospective study and found the acquired infection of *T. gondii* in patients with GTs mostly occurred within two years of diagnosis, while infection in healthy controls increases without obvious time characteristics. Chemotherapy is an important postoperative adjuvant treatment for gynecologic malignancies, patients with chemotherapy may be more susceptible to toxoplasmosis (Yu et al., 2020). Adjuvant chemotherapy is generally implemented within 3 years after surgery. This therapeutic regimen increases the risk of *T. gondii* infection. Infection of *T. gondii* in tumor patients is often ignored by clinicians, especially in patients receiving chemotherapy. Therefore, patients who received chemotherapy should be monitored for early signs of toxoplasmosis, and therapeutic prevention of *T. gondii* infection is necessary to reduce the risk of toxoplasmosis.

Our study had two main limitations. First, the study cohort was relatively small. Second, we did not collect the infection data of blood donors. Therefore, the effect caused by blood donor-derived serum antibodies was not clear.

## Conclusions

We showed the seroprevalence of *T. gondii* antibodies to be high in patients with a GT (especially for women with an ovarian cancer). The acquired infection of *T. gondii* in patients with GTs mostly occurred within two years of diagnosis. Thus, measures are needed to reduce the risk of *T. gondii* infection in patients with a GT.

## Supplemental Information

10.7717/peerj.14569/supp-1 Supplemental Information 1 Raw data.

## Additional Information and Declarations

### Data Availability

The following information was supplied regarding data availability: The raw measurements are available in the Supplemental File.
